# Survey of *Alternaria* Toxins and Other Mycotoxins in Dried Fruits in China

**DOI:** 10.3390/toxins9070200

**Published:** 2017-06-26

**Authors:** Dizhe Wei, Yao Wang, Dongmei Jiang, Xiaoyuan Feng, Jun Li, Meng Wang

**Affiliations:** 1 Beijing Research Center for Agricultural Standards and Testing, No. 9 Middle Road of Shuguanghuayuan, Haidian District, Beijing 100097, China; weidz@brcast.org.cn (D.W.); wangy@brcast.org.cn (Y.W.); jiangdm@brcast.org.cn (D.J.); fengxy@brcast.org.cn (X.F.); 2 Risk Assessment Laboratory for Agro-Products, Ministry of Agriculture, No. 9 Middle Road of Shuguanghuayuan, Haidian District, Beijing 100097, China; 3 Food Research Institute, Chinese Academy of Agricultural Sciences, No. 12 Zhongguancun South Street, Haidian District, Beijing 100081, China; lijun08@caas.cn

**Keywords:** mycotoxins, *Alternaria* toxins, dried fruits, UPLC-MS/MS, China

## Abstract

Occurrence of toxigenic molds and mycotoxins on dried fruits is a worldwide problem, but limited information is available in China. A total of 220 dried fruits (raisins, dried apricots, dates and wolfberries) purchased from China were analyzed for 17 mycotoxins (i.e., *Alternaria* toxins, ochratoxin A (OTA), patulin (PAT) and trichothecenes) by UPLC-MS/MS, combined with a single-step cleanup. The result showed that at least one mycotoxin was detected in 142 samples (64.6%). The lowest incidence of contaminated samples was observed in dried apricots (48.2%), and the highest incidence in dried wolfberries (83.3%). The *Alternaria* toxins seemed to be the major problem in dried fruits, rather than OTA or PAT. Tenuazonic acid (TeA) was the predominant mycotoxin, in both frequency and concentration, ranging from 6.9 to 5665.3 μg kg^−1^, followed by tentoxin (TEN; 20.5%), and mycophenolic acid (MPA; 19.5%). Moreover, raisins are more likely to be contaminated with OTA than the other dried fruits. Penicillic acid (PA) was detected only in dried dates, and PAT was detected only in one apricot sample. In addition, our results also showed that the simultaneous presence of 2–4 mycotoxins was observed in 31.4% of dried fruits. TeA and TEN were the most frequent combination, detected in 29 (13.2%) samples, followed by TeA and MPA with a prevalence of 11.4%. Therefore, the results of this survey suggest the need for wider monitoring on the contamination of these mycotoxins, especially *Alternaria* toxins in agro-products, and indicate the importance of setting a maximum limit for *Alternaria* toxins in China.

## 1. Introduction

Fungi belonging to the genera *Alternaria*, *Pencillium* and *Aspergillus* are the major contributors to fruit spoilage and mycotoxin production [1,2,3]. *Fusarium* species and the mycotoxins they produce (trichothecenes) were also detected in fruits [4]. In addition to these destructive pathogens causing huge economic losses, mycotoxins are of concern to human health due to their acute or chronic toxic effects, such as carcinogenic, mutagenic and teratogenic effects [2,3]. Although drying is one of the best preservation techniques for fruits, molds and mycotoxin production in dried fruits are still an important problem. This is due to the limits of drying conditions, as well as harvesting methods and sugar content or water activity in fruits. For example, dates, collected directly from the soil, favor infection risk by attack of insects and pathogens because they are outside drying longer than necessary [3]. Until now, investigations on the occurrence of mycotoxins in dried fruits have been mostly concerned with aflatoxins in figs, and OTA in dried vine fruits [5,6,7,8,9,10,11,12,13]. Therefore, to protect public health, maximum acceptable levels of mycotoxins in dried fruits have been established worldwide. European legislation is often considered as being the most comprehensive and strict in relation to mycotoxins in foods, but only aflatoxins (AFs) and ochratoxin A (OTA) in dried fruits are subject to legal guidelines. Although the *Alternaria* toxins have not been regulated, their toxigenic potential has been evaluated by the European Food Safety Authority (EFSA) [14].

*Alternaria* species can produce more than 70 toxic secondary metabolites, but only a few of them have been structurally identified and reported as mycotoxins [14]. The *Alternaria* toxins, mainly including alternariol (AOH), alternariol monomethyl ether (AME), tenuazonic acid (TeA), tentoxin (TEN) and altenuene (ALT), are widely investigated in fruit juices, tomato pastes and wines [15,16,17,18,19]. These surveys suggested that TeA frequently occurred in higher concentration compared to AOH and AME. AOH and AME, on the other hand, are possibly genotoxic [14]. EFSA also recommended their monitoring in national surveys. To the best of our knowledge, no information in the literature is available on the occurrence of *Alternaria* toxins in dried fruits.

Determination of *Alternaria* toxins and other mycotoxins in dried fruits can be an extremely challenging task due to the large quantities of co-extractive compounds, especially sugars and pigments, which can cause matrix effects and adversely affect the method performance. Recently, in our group, a sensitive and reliable method, based on a single-step SPE cleanup followed by UPLC-MS/MS, was developed for the simultaneous determination of prevalence mycotoxins in fruits [20].

In China, dried fruits (i.e., raisins, dried apricots, dates and wolfberries) may be an important dietary source of mycotoxins, since they can be used as raw material input for further applications in breakfast congee, baked goods, and tea. However, data available on the occurrence of mycotoxins in dried fruits are limited. Previous research reported only that OTA among nine target mycotoxins was the most frequent contaminant in raisins and dried dates, while trace amounts of AFB_1_ were only detected in nuts [13]. The prevalence of AFs in nuts and figs is not of our concern. Therefore, the exposure of *Alternaria* toxins and other major mycotoxins (OTA, PAT and trichothecenes) in dried fruits was investigated to reveal the real situation of contamination in China.

## 2. Results and Discussion

### 2.1. Method Validation

**Figure 1:**
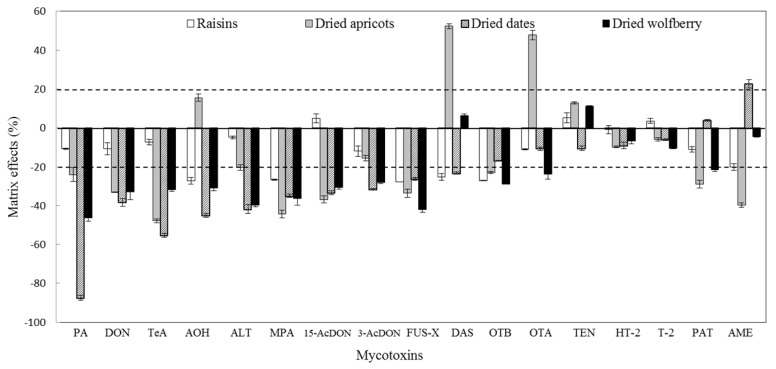
The matrix effects of 17 mycotoxins in the different dried fruits. Acceptable extents are in the range of two dashed lines (−20 to 20%) with the spiked levels of 1.0 μg kg^−1^ for OTA, OTB, AME and MPA or 10 μg kg^−1^ for AOH, TEN, ALT and T-2 or 50 μg kg^−1^ for TeA, PAT, DON, 3-AcDON, 15-AcDON, HT-2, DAS, FUS-X and PA. Vertical bars indicate ± standard errors.

For the method validation, matrix effect was considered to be a critical parameter for the correct quantification of target mycotoxins. Signal enhancement or suppression effects are considered tolerable if the value is between ±20%. To minimize the matrix effects, a rapid and simple solid phase extraction (SPE) cleanup, previously developed by our group [20], was employed in this study. Matrix effects were calculated for each mycotoxin as follows: matrix effects (%) = (1 − A_matrix_/A_standard_) × 100, where A_standard_ is the peak area of the standards in solvent and A_matrix_ is the peak area of standards in matrix-matched solution at the same concentrations. As shown in Figure 1, the slight matrix effects for target mycotoxins (−27.8% to 5.2%) could be observed in raisins, whereas dried dates showed significant signal suppression, especially for HT-2, with the largest extent being −87.5%. Moreover, the extent of matrix effects ranged from −47.9% to 27.8% for dried apricots and −46.4% to 11.1% for dried wolfberries, respectively. As a consequence, matrix-matched calibration was applied as a compromise to reduce the matrix effects, so as to guarantee a reliable quantification. After application of the matrix matched calibration curves, good linearity, satisfactory recovery and precision were obtained (Table 1).

Recovery was evaluated by spiking a known blank dried fruit sample (five replicates) at two levels (LOQ, and 10 times the LOQ). The recoveries of the 17 mycotoxins were in the range of 71.3~102.9% for raisins, 70.8~101.9% for dried dates, 68.1~111.0% for dried apricots and 69.5~104.5% for dried wolfberries, respectively (Table 1); and the RSDs for precision were in the range of 1.9~8.8%, which were both within the acceptable range recommended by the European Commission [21]. In addition, although nivalenol (NIV) is an important trichotecene from a toxicological point of view, it was out of the acceptable range recommended by EU (data not shown). Moreover, there are no reports of NIV contamination in fruits, and thus NIV was not investigated in our study. Limit of quantifications (LOQ) have been calculated according to our previous publication [20], and the values for target mycotoxins were in the range of 0.1–5.0 μg kg^−1^. The multiple reaction monitoring (MRM) chromatogram for 17 targeted mycotoxins at LOQ level is shown in Figure S1 of the supplementary materials. Compared to the previous analytical method, the method developed herein showed comparable sensitivity and accuracy values for the determination of 17 mycotoxins in dried fruits, and even better recoveries and significantly lower matrix effects for OTA in raisins [13,22]. Therefore, the current analytical method could be applied for simultaneous determination of 17 mycotoxins in dried fruits.

### 2.2. Occurrence of the Mycotoxins in Dried Fruits

Results of mycotoxin contamination in dried fruit samples are presented in Table 2, and the chromatograms of naturally contaminated dried fruit samples are shown in Figure S2 of the supplementary materials. All of the targeted mycotoxins were detected in some of the analyzed samples, except trichothecene mycotoxins (DON, Fus-X, 3-AcDON, 15-AcDON, DAS, HT-2 and T-2). The absence of trichothecene in dried fruits was also reported by the previous studies [5,7,13]. Ochratoxins were detected only in raisins (13/57) and penicillic acid (PA) was detected only in dried dates (14/53). Only one dried apricot sample was contaminated with PAT (30.6 μg kg^−1^). Mycophenolic acid (MPA) is an immunosuppressive compound with antimicrobial activities. Consumption of immunosuppressive compounds can increase the risk of infectious diseases and the toxicity of other toxins [23]. MPA was detected in all dried fruits except wolfberries, with an incidence of 19.5%.

The most prevalent mycotoxins in dried fruits were *Alternaria* toxins. The *Alternaria* toxins were all found at a considerable level except ALT. ALT was not detected in any dried fruit samples, while AOH was detected in 2.3% of the samples, AME in 8.2% of the samples and TEN in 20.5% of the samples, mainly in dried wolfberries (Table 2). AME was found more frequently than AOH as formerly reported [14,19]. TeA (42.7%) was the most frequently occurring toxin and was quantified in all dried fruits with the concentration levels in the range of 6.9–5665.3 μg kg^−1^ (Table 2). However, no literature is available on the occurrence of *Alternaria* toxins in dried fruits analyzed in this study, although *Alternaria* strains (*A. alternate*, *A. tenuissima*, *A. solani*) were isolated from fruits in several studies [1,24]. Most of the investigations on *Alternaria* toxins in fruit-derived products were focused on the analysis of fruit juices and wines [15,16,17,18,19]. The contamination trends of the four *Alternaria* toxins in dried fruits reported here are in agreement with the above surveys, in which AOH, AME, TeA and TEN were generally detected and TeA was the most widely occurring toxin. The highest incidence of TeA was found in dried wolfberries (64.8%), followed by dried apricots (37.5%), raisins (35.1%) and dried dates (34.0%). Up to now, no maximum levels have been set for *Alternaria* toxins, probably due to limited data available on their toxicity and occurrence.

#### 2.2.1. Raisins

Grapes are susceptible to fungal infection, and thus many studies have been conducted on grape-derived products to check if they had been contaminated by mycotoxins, mainly by OTA. Indeed, our results showed that OTA contamination was only found in raisin samples (Table 3). Eleven out of 57 (19.3%) dried grapes contained OTA in the range of 0.17~8.8 μg kg^−1^, with a mean value of 1.9 μg kg^−1^. None of the samples exceeded the maximal level set by the EU commission (10 μg kg^−1^). However, Han et al. [13] reported even higher levels of OTA contamination (56.5%) with a range of 0.4~65.7 μg kg^−1^ in China, and five out of 32 raisin samples exceeded the EU limit (10 μg kg^−1^). The reason for these results may be associated with various climate conditions during harvest. Meyvaci et al. [8] pointed out that the OTA occurrence in sultana samples showed significant yearly variations. Palumbo et al. [12] also reported that the occurrence of OTA-contaminated raisins ranged from 17% incidence in 2012, to 88% incidence in 2013 to 2014. Furthermore, it is the first investigation of the OTB contamination in dried vine fruits. OTB was detected only in two samples and co-occurred with OTA, but in much lower amount (0.13~0.33 μg kg^−1^).

In addition to ochratoxins, MPA and *Alternaria* toxins (AOH, AME and TeA) were detected in raisins. MPA-contaminated raisins had the highest incidence (47.4%) and the highest levels (2647.3 μg kg^−1^) in the presented study, which further confirmed the results of wine reported by Pizzuttia et al. [25]. Our results also showed that the co-occurrence of MPA with OTA and *Alternaria* toxins was detected in seven and 14 samples, respectively.

With regard to the contamination of the *Alternaria* toxins in raisin samples, AOH, AME and TeA were detected, and 24.6% of the samples showed contamination with one analyte, 12.3% were positive for two toxins and 3.5% contained three toxins. TeA was the predominant *Alternaria* toxin and co-occurred with OTA in four samples (7.0%). Moreover, TeA occurred more frequently than OTA, indicating that the TeA producing fungi, especially *Alternaria alternata* [24], could easily invade the dried vine fruits. In addition, the increase of TeA in raisin samples was often accompanied by the decline of OTA. A possible explanation might be associated with the interactions between toxin producing fungi. It was reported that the increase of *Alternaria* toxins (AOH, AME and TeA) coincided with degrading of *Fusarium* mycotoxins when *Alternaria* strains grew in wheat kernels spiked with *Fusarium* mycotoxins [26].

#### 2.2.2. Dried Apricots

In the present study, only five mycotoxins were detected in the analyzed dried apricot samples (Table 3). Unlike the results obtained in raisins, TeA was the most prevalent mycotoxin, followed by MPA. Twenty-one out of 56 (37.5%) dried apricots were contaminated with a mean level of 237.1 μg kg^−1^ TeA. Six samples (10.8%) contained TeA at a concentration higher than 300 μg kg^−1^, and one of them reached 1231.8 μg kg^−1^. However, AOH, found in raisins, was not a contaminant in dried apricots; the other two *Alternaria* toxins, TEN (4/56) and AME (3/56), were sporadically detected in the samples. This may be due to the different toxigenic profiles by *Alternaria* species. As reported previously, 53% of *Alternaria alternata* isolated from wine grapes could co-produce the three mycotoxins (TeA, AOH and AME), while TeA was the toxin produced at highest frequency (97%) and at highest levels [27]. It was also reported that *A. tenuissima* and *A. arborescens* isolated from Greek apples could produce AOH, AME and TEN, but *A. arborescens* produced more TEN than *A. tenuissima* [28]. In addition, apricots were also considered as suitable substrates for PAT production [1]. This view is further supported by our study. PAT was only detected in dried apricots, and a single sample was contaminated with 30.6 μg kg^−1^ of PAT.

**Figure 2:**
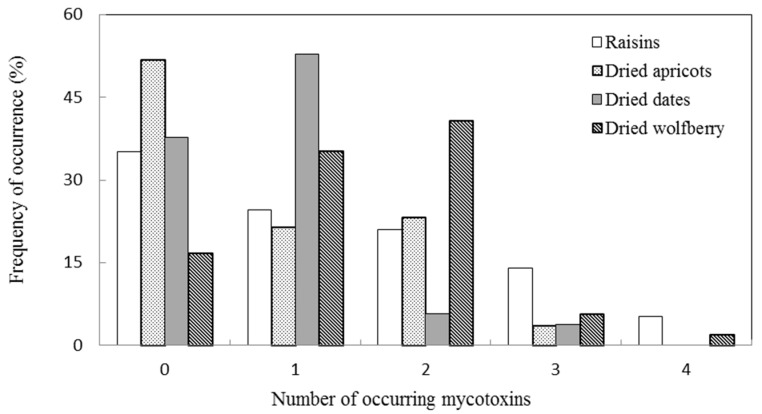
Frequency of occurrence (%) of mycotoxins in the different dried fruits collected from retail outlets in Beijing, China.

There are few reports regarding the natural occurrence of mycotoxins in dried apricots, including the contamination of aflatoxins, OTA and emerging *Fusarium* mycotoxins [6,9,10,11]. In good agreement with our results, none of the dried apricots analyzed in Brazil [9] and Spain [11] were contaminated with detectable levels of OTA. On the other hand, low incidence and concentration of OTA were found in the samples analyzed in Turkey [10] and Tunisia [11]. It is worth noting that 51.8% of apricot samples were free from detectable levels of the target mycotoxins (Figure 2). The low incidence of mycotoxin contamination in dried apricots could be a result of sulfur dioxide treatment, commonly used in drying [2].

#### 2.2.3. Dried Dates

Dates are grown under high humidity and moderate temperatures, so they easily suffer from mycotoxin contamination [3]. As shown in Table 3, two *Alternaria* toxins (TeA and TEN), PA and MPA were detected in date samples. Similar to the result obtained in dried apricots, TeA was the most prevalent toxin (34.0%) with the highest concentration. Eight samples (15.1%) contained TeA at a concentration higher than 1000 μg kg^−1^, and up to 4411.4 μg kg^−1^ was detected in one of the samples. Meanwhile, the highest mean value of TeA among the dried fruits in this study was detected in dried dates. To the best of our knowledge, the natural occurrences of *Alternaria* toxins in dried dates was reported for the first time, and the results suggested a high incidence; therefore, more occurrence data are necessary to properly evaluate the exposure of TeA through the consumption of dates.

In addition to *Alternaria* toxins, 14 out of the 53 samples were contaminated with PA ranging from 20.4 to 85.6 μg kg^−1^, indicating that the *Penicillium* or *Aspergillus* species, potential producers of PA [29], are prevalent in dates. PA is cytotoxic to plant and animal cells and has also been reported as genotoxic to microorganisms [30], but scarce literature is available on the occurrence of PA in foods, probably as a result of its inherent instability. Similar to PAT, the unsaturated lactone system reacts readily with sulfhydryl compounds, such as glutathione and cystein, and is rapidly converted to other products, thereby losing its biological activity [31]. With respect to OTA, numerous surveys reported a low incidence (2.4–9.0%) of OTA contamination in dried dates [9,12]. However, no OTA was detected in our study. The relatively low contamination levels of OTA reported here may be due to the different harvest date, drying process, or preharvest factors such as the temperature and humidity of the surroundings. The previous finding suggested that the late-maturing varieties of dates had less mycotoxin contamination than early-maturing varieties [32]. Removal of decayed or damaged dates before drying may also reduce the incidence of mycotoxin contamination.

#### 2.2.4. Dried Wolfberries

Dried wolfberries (*Lycium barbarum* L.) have been used as a traditional Chinese medicine for more than 2000 years due to their health benefits for livers and kidneys. However, our results have shown that the highest contamination incidence was observed in dried wolfberries compared with the other three dried fruits (Table 3). None of the targeted mycotoxins was detected only in 16.7% (9/54) of the dried wolfberry samples (Figure 2). As reported previously, wolfberry fruit rots very easily in the drying process, especially in the drying by insolation method. The rotting rate even reaches up to 60~70%, and *Alternaria alternata* is the main latent invading pathogenic fungi [33]. This view is further supported by the present study. Only *Alternaria* toxins were detected in dried wolfberries, of which TeA (64.8%) and TEN (63.0%) were the most prevalent toxins (Table 3). Further, wolfberry samples contained the highest levels of TeA (max: 5665.3 μg kg^−1^), TEN (max: 1032.6 μg kg^−1^), AOH (max: 27.4 μg kg^−1^) and AME (max: 15.0 μg kg^−1^) among all dried fruits in the present study. Even the TEN and TeA levels exceeded 1000 μg kg^−1^ in one (1.9%) and six (11.1%) samples, respectively. In addition, the concentration of TEN was higher than that of TeA in 40.7% of wolfberries. It is the first time that mycotoxins in dried wolfberries have been detected, indicating that this popular food has potential health risks to consumers due to its mycotoxin contamination, and thus should be brought into the monitoring systems. Meanwhile, further studies are necessary to clarify the behavior of *Alternaria* toxins during storage and drying.

### 2.3. Co-Occurrence of Mycotoxins

Overall, 1.8% (4/220) of dried fruits was contaminated simultaneously by four toxins, 6.8% (15/220) by three toxins, 22.7% (50/220) by two toxins and 33.2% (73/220) by only one mycotoxin. Regarding the co-occurrence of two toxins, TeA and TEN was the most frequent combination detected in 29 (13.2%, 29/220) samples due to the high co-contamination in dried wolfberries, followed by TeA and MPA with the prevalence of 11.4% (25/220). Depending on the matrix, the co-occurrence of mycotoxin in dried dates was occasional; only five samples (9.4%) were co-contaminated with two or three mycotoxins. However, a co-occurrence with at least two mycotoxins was detected in 40.4% (23/57) of raisins or 48.2% (26/54) of dried wolfberries. It may be associated with the higher number (six) of mycotoxins detected in raisin samples, but no more than four mycotoxins were simultaneously detected. In terms of dried wolfberries, the high incidence of two *Alternaria* toxins was observed, indicating matrix may influence the toxin production of *Alternaria* species, which is in accordance with previous studies [28]. The simultaneous presence of four *Alternaria* toxins took place only in one sample.

## 3. Conclusions

A reliable method for screening of 17 mycotoxins (i.e., *Alternaria* toxins, OTA, PAT and trichothecenes) was developed and validated in dried fruits, using ultra high performance liquid chromatography coupled to tandem mass spectrometry, combined with a single-step cleanup. To the best of our knowledge, no information is available on the occurrence of *Alternaria* toxins in dried fruits. The results revealed a non-negligible contamination with *Alternaria* toxins, especially in dried wolfberries, which contained the highest levels of four *Alternaria* toxins (TeA, TEN, AOH and AME) among the dried fruits studied. TeA was the predominant mycotoxin detected, by either frequency or concentration, in dried apricots, dates and wolfberries, while in raisins MPA is the prevalent mycotoxin and can occur at high levels, followed by TeA. The lowest incidence of mycotoxin contamination was observed in dried apricots. In addition, raisins are more likely to be contaminated with OTA than are other dried fruits. PA was detected only in dried dates and PAT was detected only in one apricot sample. Furthermore, our results also showed that mycotoxin co-occurrence in raisin and dried wolfberry was more frequent than that in dried date and apricot. Therefore, there is a need for more occurrence data to estimate the exposure to mycotoxins, especially *Alternaria* toxins, from fruit-based products in Chinese populations.

## 4. Materials and Methods

### 4.1. Reagents

Acetonitrile (MeCN), methanol (MeOH), formic acid and ammonium acetate (NH_4_AC) of MS grade were purchased from Thermo Fisher Scientific (Fair Lawn, NJ, USA). Ultrapure water was obtained by a Milli-Q-System (Millipore, Bedford, MA, USA). Citric acid and sodium chloride (NaCl) were of analytical grade. Aminopropyl (-NH_2_) and mixed-mode cationic exchange (MCX) sorbents with 40~60 μm of particle size were obtained from Guangpuda Technologies (Beijing, China).

### 4.2. Standards

The certified standards of altenariol (AOH), altenariol monomethyl ether (AME), tentoxin (TEN), tenuazonic acid (TeA), ochratoxin A (OTA), OTB, patulin (PAT), diacetoxyscirpenol (DAS), deoxynivalenol (DON), 3-acetyl deoxynivalenol (3-AcDON), 15-AcDON, fusarenon-X (FUS-X), T-2 and HT-2 toxin were purchased from Romer Labs Inc. (Union, MO, USA). Altenuene (ALT) was purchased from Toronto Research Chemicals Inc. (Toronto, ON, Canada). Mycophenolic acid (MPA) and penicilic acid (PA) were acquired from Fermenteck Ltd. (Jerusalem, Israel).

The individual stock solutions of OTA, OTB, AME and MPA at 10 mg L^−1^ were prepared in MeCN, whereas the rest of compounds were prepared at 100 mg L^−1^ in MeCN. An intermediate mixed solution (MIX A) containing OTA, OTB, AME and MPA at a concentration of 0.1 mg L^−1^ was obtained after mixing individual stock solutions and diluting with MeCN. A similar procedure was carried out with AOH, TEN, ALT and T-2 toxin, at a concentration of 1 mg L^−1^ (MIX B). In the same way, TeA, PAT, DON, 3-AcDON, 15-AcDON, HT-2, DAS, FUS-X and PA were mixed in order to obtain a concentration of 5 mg L^−1^ (MIX C). The total mixed standard solution was prepared by adding 1 mL of MIX A, 1 mL of MIX B, and 1 mL of MIX C, and diluting to 10 mL with MeCN. Working standard solutions for UPLC-MS/MS analysis and for fortification of samples were prepared by dilution of the total mixed standard solution with water and were stored in darkness at −20 °C for one month. The spiking solution was freshly prepared from the total mixed solutions, and the working solutions with different concentrations were freshly prepared just before use with the blank matrix.

### 4.3. Samples

A total of 220 samples consisting of raisins (57), dried apricots (56), dried dates (53) and dried wolfberries (54) were randomly collected from different supermarkets and local markets in Beijing, China, during 2016~2017. All the products were from main production regions of the dried fruits, principally Xinjiang province for raisins, dried apricots and dates, and Ningxia province for dried wolfberries; and a few “other” regions where a very small number of samples were collected. The collected dried fruits were prepared according to previous studies [11,13]: the samples were cut into small pieces, homogenized and milled. All samples were stored in polyethylene bags and maintained at refrigeration temperature (4 °C) in a dark and dry place until analysis.

### 4.4. Sample Preparation

#### 4.4.1. Extraction

All samples were analyzed for 17 mycotoxins based on the methods reported previously by Wang et al. [20] with minor modification. Briefly, an aliquot of 5.0 g dried fruits was mixed with 5 mL of 50 mM citric acid in a 50 mL polypropylene centrifuge tube. Then, 20 mL acetonitrile was added and the mixture was homogenized with a high speed blender (Ultra-Turrax T25, IKA, Staufen, Germany) for 3 min. After the addition of 2 g NaCl, the mixture was shaken vigorously for 1 min and centrifuged at 10,000 rpm for 5 min at 10 °C.

#### 4.4.2. Cleanup

SPE cartridges were prepared according to our previous publication [20]. A 4.0 mL aliquot of upper MeCN layer was passed through the homemade SPE (MCX + NH_2_) cartridge and collected. Finally, the cleanup extract was evaporated to dryness at 50 °C under a gentle nitrogen stream, and reconstituted with 1 mL of MeCN/water (3:7, *v*/*v*) containing 1 mM NH_4_AC, and then the obtained solution was forced through a 0.22 μm PTFE membrane filter (Pall, MI, USA). The supernatant was analyzed for the 17 mycotoxins by UPLC-MS/MS.

### 4.5. UPLC-MS/MS Analysis

A UPLC^TM^ system (Acquity, Waters, Milford, MA, USA) was interfaced to a triple quadrupole mass spectrometer (TQ-S, Waters Micromass, Manchester, UK) using an orthogonal Z-spray electrospray ionization (ESI) interface. The LC separation was performed using an Acquity Cortecs UPLC C18 column (1.6 μm particle size; 2.1 × 100 mm, Waters, Milford, MA, USA), maintaining the column temperature at 40 °C. The mobile phase consisted of water containing 1 mM NH_4_AC (A) and MeOH (B). The separation was performed at a flow rate of 0.3 mL min^−1^, with a gradient elution starting at 5% of phase B and held for 0.5 min, rising linearly to 90% phase B over 6.5 min and the column was washed for 0.5 min with 90% organic phase (B). Then, the mobile phase composition was returned to the initial condition in 0.1 min and this composition was held for 1.4 min for re-equilibration, resulting in a total run time of 9 min.

Based on the structural properties of analytes, both the positive and negative ionization modes were applied. The parameters were as follows: capillary voltage, +2.5 kV/−1.0 kV; source temperature, 150 °C; desolvation temperature, 400 °C; cone gas flow, 150 L h^−1^; and desolvation gas flow, 800 L h^−1^. Detection was carried out in multiple reactions monitoring (MRM) mode. All analyte dependent parameters are summarized in Table S1 of the supplementary materials. The MassLynx^TM^ 4.1 software (Waters, Milford, MA, USA) was used for data acquisition and processing. A toxin-free dried fruit sample was used as a base for the matrix-matched calibration standards for quantification. In terms of the matrix-matched calibration, the final concentration of AME, TeA, TEN and MPA in some samples is exceeded. Therefore, the further dilution with blank matrix was performed until the final concentration of target mycotoxins was in the linear calibration range.

### 4.6. Method Validation

Method validation was carried out according to European guideline SANTE/11945/2015 [21] for determination of the following parameters: linearity, selectivity, matrix effects, accuracy (recovery), precision (%RSD), as well as limits of quantifications (LOQ). Selectivity was checked looking at any interference peak in the MS/MS transition at the retention time of each mycotoxin. The LOQ was based on the signal to noise observed in a sample spiked at 0.1 ng mL^−1^ for group Mix A, or in a sample spiked at 1 ng mL^−1^ for group Mix B, or in a sample spiked at 5 ng mL^−1^ for group Mix C showing a lower response. Recoveries and precision were determined intra-day by analyzing spiked blank samples in five replicates at two levels: at the LOQ and at 10 times the LOQ. Spiked samples were extracted and analyzed using the same UPLC-MS/MS conditions as described above. Analytical recovery was calculated by comparison with matrix-matched standard calibrations. The matrix effects were evaluated according to the following formula: the matrix effects (%) = [(the response of the target compound in matrix − the response of the target compound in solvent)/the response of the target compound in solvent] × 100%.

## Figures and Tables

**Table 1:** Validation parameters of the proposed UPLC-MS/MS method for 17 target mycotoxins in dried fruits.

Mycotoxins ^a^	Linear Range (ng mL^−1^)	LOQ (ng mL^−1^) ^b^	Spiked Levels	Recovery ± RSD (%) ^c^			
				Raisins	Dried Apricots	Dried Dates	Dried Wolfberries
AME	0.1~10	0.1	LOQ	85.2 ± 2.8	87.9 ± 4.3	83.1 ± 3.0	96.6 ± 2.6
			10 LOQ	96.8 ± 3.8	101.3 ± 2.7	102.3 ± 2.2	98.7 ± 2.2
PAT	5~500	5	LOQ	82.0 ± 3.0	76.2 ± 6.2	87.8 ± 3.4	73.0 ± 6.4
			10 LOQ	91.5 ± 3.6	86.1 ± 3.5	96.8 ± 4.7	86.1 ± 4.3
AOH	1~100	1	LOQ	91.1 ± 2.4	95.9 ± 2.9	89.0 ± 3.8	80.5 ± 3.0
			10 LOQ	93.4± 2.8	97.0 ± 3.8	101.1 ± 3.1	96.9 ± 5.4
TeA	5~500	5	LOQ	82.9 ± 4.1	91.2 ± 1.9	82.3 ± 6.6	90.6 ± 3.6
			10 LOQ	90.0 ± 3.8	100.1 ± 4.9	101.9 ± 3.6	102.9 ± 6.2
TEN	1~100	1	LOQ	89.1 ± 3.2	85.9 ± 4.5	93.4 ± 4.6	89.8 ± 3.6
			10 LOQ	102.5 ± 2.8	104.2 ± 3.4	106.6 ± 5.1	104.5 ± 5.6
ALT	1~100	1	LOQ	84.4 ± 5.1	90.1 ± 3.8	89.4 ± 6.7	92.3 ± 2.3
			10 LOQ	95.8 ± 2.2	99.1 ± 4.1	98.8 ± 5.6	100.8 ± 2.6
OTA	0.1~10	0.1	LOQ	85.5 ± 4.3	95.6 ± 4.8	80.3 ± 4.7	83.3 ± 3.9
			10 LOQ	97.7 ± 3.1	111.0 ± 3.7	92.7 ± 2.8	87.0 ± 2.4
OTB	0.1~10	0.1	LOQ	90.6 ± 4.7	86.7 ± 4.3	82.1 ± 2.4	80.6 ± 2.6
			10 LOQ	90.7 ± 4.2	107.1 ± 3.0	95.8 ± 3.6	96.0 ± 4.5
PA	5~500	5	LOQ	94.7 ± 5.6	82.4 ± 5.9	95.3 ± 4.1	89.4 ± 2.8
			10 LOQ	96.1 ± 3.2	81.2 ± 6.9	98.4 ± 3.3	92.1 ± 4.4
DON	5~500	5	LOQ	71.3 ± 6.2	68.1 ± 6.4	70.8 ± 5.1	69.5 ± 5.9
			10 LOQ	75.1 ± 4.1	70.6 ± 5.8	75.3 ± 3.8	72.4 ± 4.2
Fus-X	5~500	5	LOQ	82.9 ± 4.8	83.5 ± 5.3	76.7 ± 4.2	83.8 ± 4.0
			10 LOQ	85.6 ± 3.0	88.8 ± 4.8	85.8 ± 5.8	90.8 ± 3.3
3-AcDON	5~500	5	LOQ	84.8 ± 6.6	84.9 ± 6.5	83.6 ± 8.8	83.4 ± 4.6
			10 LOQ	91.4 ± 3.5	92.4 ± 4.5	94.3±4.3	89.7 ± 3.2
15-AcDON	5~500	5	LOQ	88.1 ± 5.5	87.7 ± 7.6	89.6 ± 7.5	85.3 ± 6.0
			10 LOQ	90.9 ± 6.8	95.6 ± 4.3	96.0 ± 3.7	87.0 ± 5.0
DAS	5~500	5	LOQ	88.1 ± 4.3	77.8 ± 8.1	90.2 ± 5.8	75.9 ± 7.0
			10 LOQ	90.7± 5.2	89.1 ± 2.9	91.0 ± 5.0	87.1 ± 5.6
HT-2	5~500	5	LOQ	85.6 ± 3.1	75.4 ± 7.4	78.6 ± 8.2	77.3 ± 6.7
			10 LOQ	89.3 ± 4.9	87.1 ± 4.0	90.3 ± 7.8	83.3 ± 4.4
T-2	1~100	1	LOQ	95.4 ± 4.0	91.9 ± 5.5	99.3 ± 4.1	95.4 ± 6.6
			10 LOQ	100.6 ± 3.8	97.9 ± 3.4	101.8 ± 5.1	100.9 ± 4.3
MPA	0.1~10	0.1	LOQ	93.9 ± 4.9	90.7 ± 4.2	92.8 ± 3.7	88.8 ± 4.4
			10 LOQ	102.9 ± 3.3	99.3 ± 3.3	100.6 ± 4.4	96.6 ± 5.4

^a^ AME: alternariol monomethyl ether; PAT: patulin; AOH: alternariol; TeA: tenuazonic acid; TEN: tentoxin; ALT: altenuene; OTA: ochratoxin A; OTB: ochratoxin B; PA: penicillic acid; DON: deoxynivalenol; Fus-X: fusarenon-X; 3-AcDON: 3-acetyl-deoxynivalenol; DAS: diacetoxyscirpenol; MPA: mycophenolic acid. ^b^ LOQ: limit of quantification. ^c^ For each concentration level, mean recovery and RSD were calculated on *n* = 5.

**Table 2:** Mycotoxins detected in the dried fruits, specifying the number of positive samples, their occurrence, mean, median and range.

Mycotoxins	Positives (N)	Occurrence (%)	Mean (μg kg^−1^)	Median (μg kg^−1^)	Range (μg kg^−1^)
AME	18	8.2	3.0	0.5	0.2~15.0
PAT	1	0.5	30.6	30.6	30.6
AOH	5	2.3	12.0	7.5	3.5~27.4
TeA	94	42.7	456.5	83.5	6.9~5665.3
TEN	45	20.5	120.5	60.8	1.4~1032.6
OTA	11	5.0	1.9	0.4	0.2~8.8
OTB	2	0.9	0.2	0.2	0.1~0.3
PA	14	6.3	39.1	35.2	20.4~85.6
MPA	43	19.5	91.6	5.5	0.3~2647.3

**Table 3:** Mycotoxins detected in raisins, dried apricots, dates and wolfberries, specifying the number of positive samples, their occurrence, mean, median and range.

Mycotoxins	Positives (N)	Occurrence (%)	Mean (μg kg^−1^)	Median (μg kg^−1^)	Range (μg kg^−1^)
Raisins					
TeA	20	35.1	104.8	29.4	6.9~594.4
AOH	3	5.3	8.9	7.5	3.5~15.6
AME	11	19.3	3.1	0.4	0.3~13.5
OTA	11	19.3	1.9	0.4	0.2~8.8
OTB	2	3.5	0.2	0.2	0.1~0.3
MPA	27	47.4	130.7	9.4	0.3~2647.3
Dried apricots					
TeA	21	37.5	237.1	71.8	10.4~1231.8
TEN	4	7.1	14.0	12.6	2.7~28.0
AME	3	5.4	1.3	1.2	0.5~2.1
PAT	1	1.8	30.6	30.6	30.6
MPA	15	26.8	26.7	3.9	1.0~119.7
Dried dates					
TeA	18	34.0	873.2	555.0	9.6~4411.4
TEN	7	13.2	6.2	5.2	1.4~11.2
PA	14	26.4	39.1	35.2	20.4~85.6
MPA	1	1.9	7.5	7.5	7.5
Dried wolfberries					
TeA	35	64.8	574.8	93.4	23.8~5665.3
TEN	34	63.0	156.5	75.9	11.7~1032.6
AOH	2	3.7	16.6	16.6	5.9~27.4
AME	4	7.4	3.9	0.3	0.2~15.0

## Supplementary Materials

The following are available online at www.mdpi.com/2072-6651/9/7/200/s1, Figure S1: MRM chromatograms for 17 targeted mycotoxins at a LOQ level validated in raisins, Figure S2: MRM chromatograms of naturally contaminated samples, Table S1: Optimized MRM parameters for mycotoxin analyzed.
