# Supplementary material for: Survey of Alternaria Toxins and Other Mycotoxins in Dried Fruits in China
Source: Toxins (Basel). 2017 Jun 26;9(7):200. doi: 10.3390/toxins9070200 (PMC5535147; doi:10.3390/toxins9070200)
Supplement: Supplementary file 1 [file toxins-09-00200-s001.pdf]

Supplementary Materials: Survey of *Alternaria* Toxins and Other Mycotoxins in Dried Fruits in China

Dizhe Wei, Yao Wang, Dongmei Jiang, Xiaoyuan Feng, Jun Li and Meng Wang

Table S1. Optimized MRM parameters for mycotoxin analyzed.

Mycotoxins[a]	Ionization mode	Retention Time (min)	Precursor ion (m/z)	Quantitative ion (Collision Energy)	Qualitative ion (Collision energy)	Cone voltage (V)
AME	ESI-	6.85	271.0	256.0 (35)	213.0 (20)	32
PAT	ESI-	2.12	153.0	108.9 (10)	80.9 (12)	10
AOH	ESI+	5.74	259.0	213.2 (20)	185.1 (30)	30
TeA	ESI+	2.45	198.2	125.0 (20)	139.0 (15)	24
TEN	ESI+	6.02	415.2	312.1 (20)	171.5 (20)	17
ALT	ESI+	5.10	293.1	257.0 (14)	239.1 (20)	16
OTA	ESI+	5.54	404.2	239.1 (19)	358.1 (14)	30
OTB	ESI+	4.96	370.2	205.2 (22)	187.1 (36)	32
PA	ESI+	2.92	171.1	125.1 (12)	153.1 (7)	20
DON	ESI+	2.93	297.1	102.0(15)	249.2 (10)	30
Fus-X	ESI+	3.57	355.2	247.1 (15)	229.1 (10)	28
3-AcDON	ESI+	4.22	339.2	231.1 (14)	203.0 (12)	28
15-AcDON	ESI+	4.24	339.2	261.1 (10)	137.0 (20)	30
DAS	ESI+	5.22	367.2	307.2 (10)	289.1 (12)	20
HT-2	ESI+	5.87	442.2	263.1 (20)	105.1 (37)	25
T-2	ESI+	6.28	484.2	305.1 (8)	245.1 (9)	30
MPA	ESI+	5.04	321.0	207.0 (24)	159.0 (30)	22

[a] AME: alternariol monomethyl ether; PAT: patulin; AOH: alternariol; TeA: tenuazonic acid; TEN: tentoxin; ALT: altenuene; OTA: Ochratoxin A; OTB: Ochratoxin B; PA: Penicilic acid; DON: deoxynivalenol; Fus-X: Fusarenon-X; 3-AcDON: 3-Acetyl-Deoxynivalenol; DAS: Diacetoxyscirpenol; MPA: Mycophenolic acid.

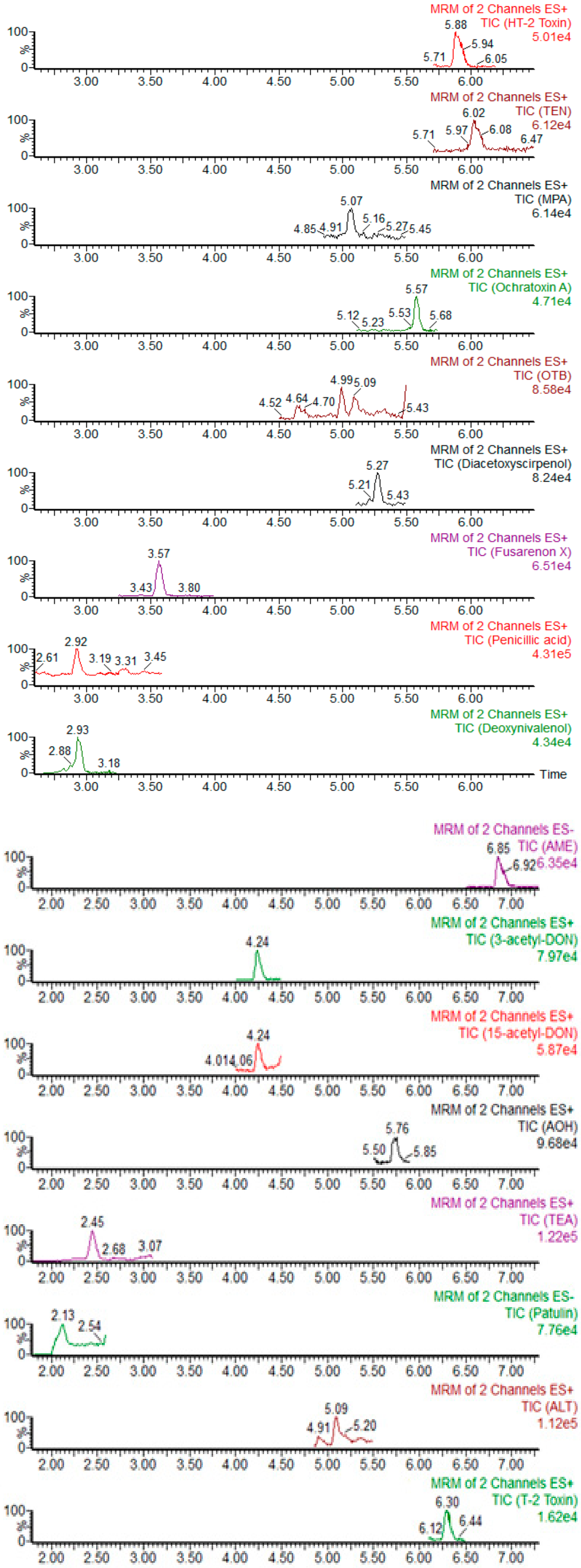

Figure S1. MRM chromatograms for 17 targeted mycotoxins at a LOQ level validated in raisins.

(a)

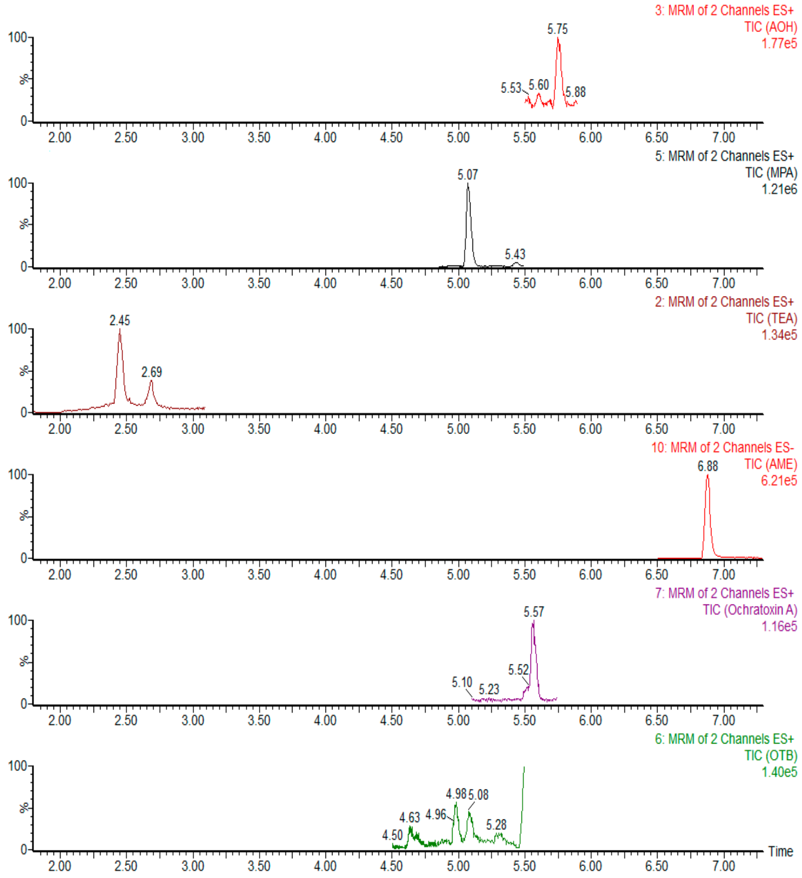
3: MRM of 2 Channels ES+
TIC (AOH)
1.77e5
5: MRM of 2 Channels ES+
TIC (MPA)
1.21e6
2: MRM of 2 Channels ES+
TIC (TEA)
1.34e5
10: MRM of 2 Channels ES-
TIC (AME)
6.21e5
7: MRM of 2 Channels ES+
TIC (Ochratoxin A)
1.16e5
6: MRM of 2 Channels ES+
TIC (OTB)
1.40e5
Time

(b)

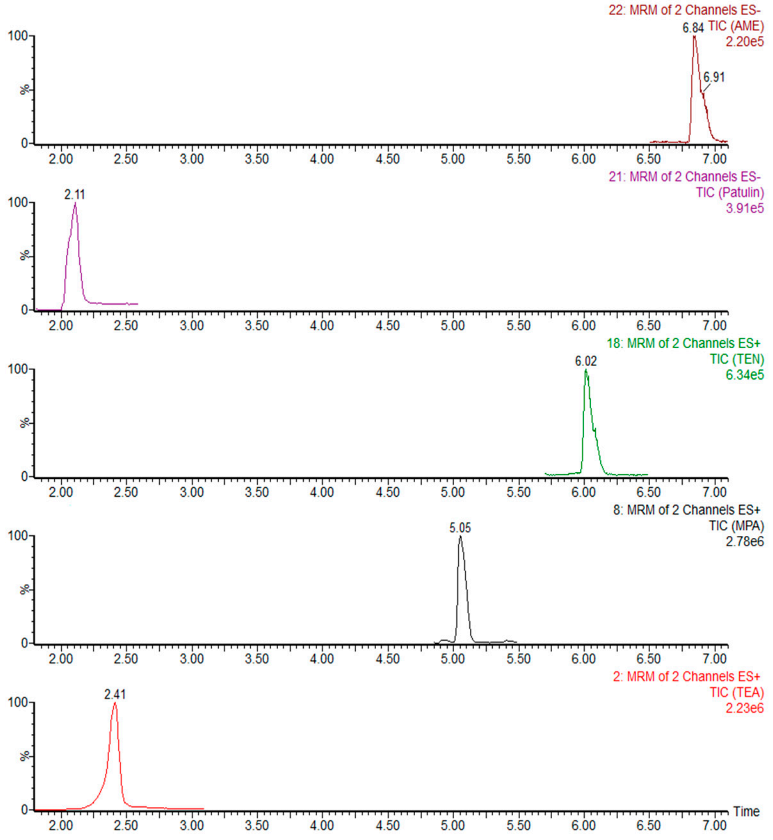
22: MRM of 2 Channels ES-
TIC (AME)
2.20e5
21: MRM of 2 Channels ES-
TIC (Patulin)
3.91e5
18: MRM of 2 Channels ES+
TIC (TEN)
6.34e5
8: MRM of 2 Channels ES+
TIC (MPA)
2.78e6
2: MRM of 2 Channels ES+
TIC (TEA)
2.23e6
Time

(c)

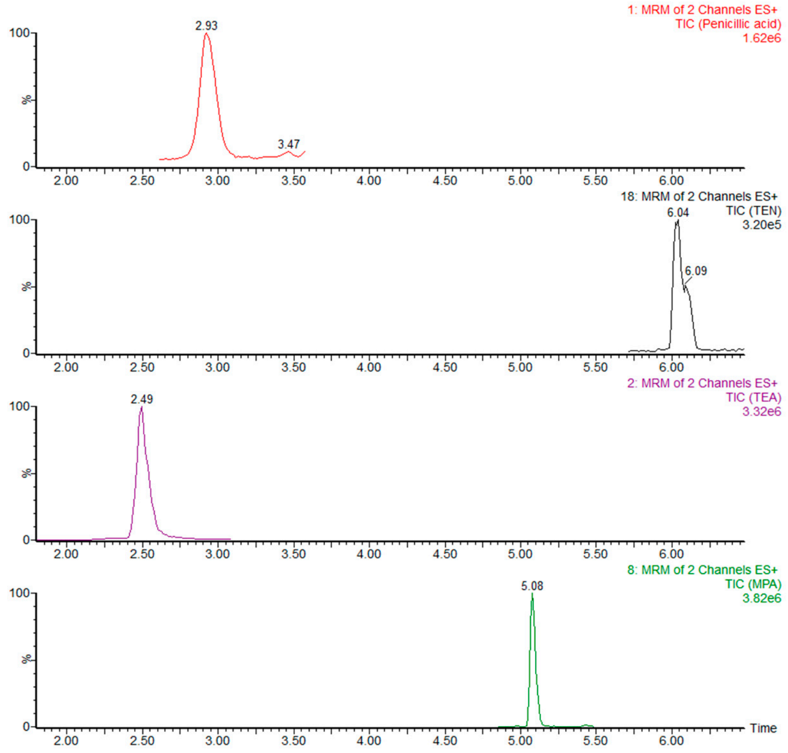

(d)

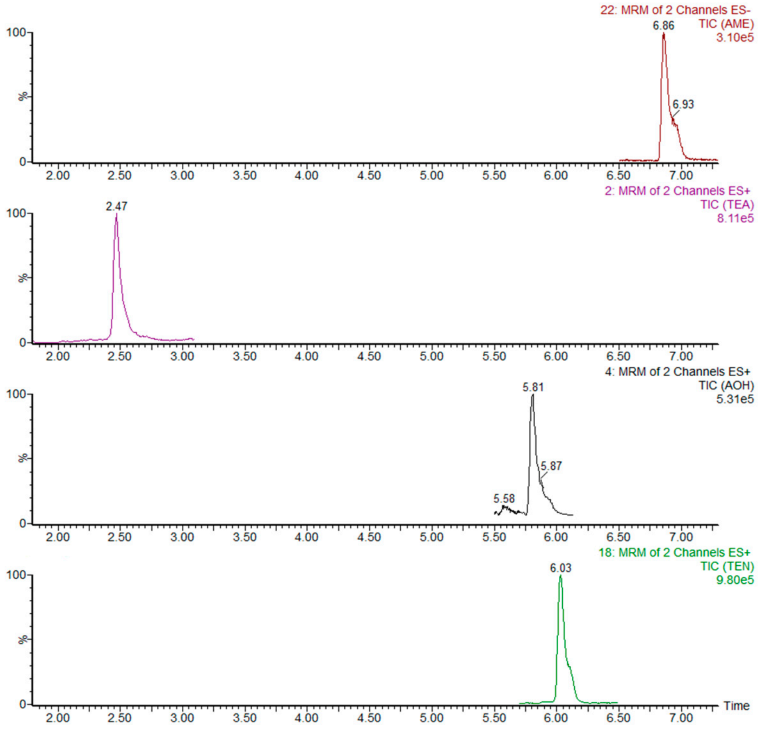

Figure S2. MRM chromatograms of naturally contaminated samples. (a) raisins with 1.8 ng mL^{-1} for AOH, 2.0 ng mL^{-1} for MPA, 5.5 ng mL^{-1} for TeA; 1.0 ng mL^{-1} for AME, 0.2 ng mL^{-1} for OTA and 0.1 ng mL^{-1} for OTB; (b) dried apricots with 0.2 ng mL^{-1} for AME, 30.6 ng mL^{-1} for PAT, 10.3 ng mL^{-1} for TEN, 5.0 ng mL^{-1} for MPA and 100.5 ng mL^{-1} for TeA; (c) dried dates with 20.4 ng mL^{-1} for PA, 5.0 ng mL^{-1} for TEN, 145.6 ng mL^{-1} for TeA and 7.5 ng mL^{-1} for MPA; (d) dried wolfberries with 0.6 ng mL^{-1} for AME, 145.6 ng mL^{-1} for TeA, 5.9 ng mL^{-1} for AOH;5.0 ng mL^{-1} for TEN.
